# Community Veterinary Medicine Programs: Pet Owners' Perceptions and Experiences

**DOI:** 10.3389/fvets.2021.678595

**Published:** 2021-06-08

**Authors:** Lori R. Kogan, Veronica H. Accornero, Emily Gelb, Margaret R. Slater

**Affiliations:** ^1^Clinical Sciences Department, Colorado State University, Fort Collins, CO, United States; ^2^Strategy and Research, The American Society for the Prevention of Cruelty to Animals, New York, NY, United States; ^3^Sheltering and Veterinary Services Department, The American Society for the Prevention of Cruelty to Animals, New York, NY, United States

**Keywords:** community program, financial limitations, access, low-cost veterinary care, disadvantaged pet owners

## Abstract

Many American pet owners struggle financially, and the COVID-19 pandemic has only exacerbated this problem. Yet, the positive effects that companion animals have in people's lives create the need for supportive systems to ensure that financial limitations, and other barriers, do not preclude pet ownership. To help address these barriers, and reach underserved communities, various forms of community-based veterinary medicine programs have been developed across the country. This study assesses two community-based veterinary programs in North Carolina, USA. In addition to perceptions surrounding veterinary services, this study paid specific attention to communication and respect; two additional elements needed for successful community-based veterinary programs. Surveys were given to clients accessing Asheville Humane Society (AHS) mobile veterinary care clinic and Asheville Humane Society (AHS) Affordable Pet Care Clinic. Results of the anonymous survey indicate that the majority of clients had positive veterinary care experiences in terms of both veterinary services and client communication. In conclusion, low-cost or free community veterinary programs—with effective communication, empathy, and cultural competence—can help open the door to future positive veterinary experiences for disadvantaged pet owners.

## Introduction

It is estimated that 38% of US households include at least one dog and 25% include at least one cat (1) with the majority of these owners reporting that their pets are part of their family (2). The inclusion of pets in the household has been shown to offer numerous physical, emotional, and social benefits (3–12). Studies exploring the benefits of pets for vulnerable populations suggest similarly positive effects. Schmitz et al. (13), for example, found that LGBTQ+ young adults report that their pets play a critical role in helping them manage major life stress and depression (13). Studies involving homeless individuals have found that their pets provide them with a myriad of benefits including safety, personal responsibility and improved emotional and mental health (14–16).

A substantial number of Americans, many of which are pet owners, struggle financially; with the COVID-19 pandemic only exacerbating this problem (17–19). This means that many families are struggling to pay their bills, including the ability to afford the veterinary care their pets need to stay happy and healthy (20). Yet, the myriad of positive effects that companion animals have in people's lives suggest the need for supportive systems to ensure that financial limitations do not preclude pet ownership (21).

The inability to access veterinary care is not simply a financial problem. Numerous barriers to veterinary care have been identified and all need to be addressed in order to support vulnerable pet owners. While financial barriers are the most common (21), and create the most stress within the veterinary profession, other barriers include hours of operation, geographical location, transportation, equipment to transport pets, cultural/language related issues, and veterinarian–client communication (16, 21, 22). An unfortunate consequence of these barriers is that low income individuals in underserved communities are frequently neglected by animal care providers (21). Yet, increased veterinary care access in these underserved areas can help reduce animal overpopulation, improve animal welfare and overall community health (23).

To help address these barriers, and reach these underserved areas, many communities have implemented some form of community-based veterinary medicine programs. Community-based veterinary medicine initiatives are seen as one method to address the lack of access to veterinary care among underserved communities (23) with community veterinarians seen as pivotal players in fostering positive interactions and experienced with pet owners through education and veterinary care (24).

Despite the growing number of community-based veterinary medicine initiatives, little research has been conducted on the topic, with most published work focused on veterinarian and student opinions or client demographics. Therefore, the quality of these programs, as well as their impact on the targeted population, is largely unknown. It has been suggested that there is a need for empirical research that explores the efficacy and potential impact of these community initiatives (22). This study addresses this call by assessing two community-based veterinary programs in located in North Carolina, USA. Given the significant impact that good communication between veterinarians and pet owners can have on pet health care, this study paid specific attention to this critical element needed for successful community-based veterinary programs.

## Materials and Methods

The perceptions and experiences of all pet owners who accessed veterinary care through one of two community-based programs in North Carolina, USA were garnered through an online, anonymous survey created in Qualtrics. All participants were sent a text message asking for their participation with an accompanying URL link and two follow-up text reminders. Follow-up phone calls and phone-based surveys were offered to owners who did not respond to the text requests. The link directed them to a survey that began by explaining the purpose of the study and their rights as participants. The survey included demographic items (i.e., gender, employment status, household income, age, ethnicity, race, and language most comfortable using) and questions pertaining to the pet obtaining veterinary care. Potential barriers to care were assessed along with owners' perception of the care and communication they received during their last visit to the clinic. The perception questions were adapted from the Client Satisfaction Questionnaire (25). These questions included perceptions of respect, empathy, communication and consideration of their culture and beliefs, and asked participants to rate each item on a 3-point scale (good, neutral, and poor). Additionally, participants were asked, hypothetically, to indicate how important the received veterinary care was in helping them keep their pet using a 3-point scale (very important, moderately important, and not important). Upon completion of the survey, all participants were entered into a raffle for a local grocery store gift card. The survey was reviewed by staff members of both Asheville Humane Society and The American Society for the Prevention of Cruelty to Animals (ASPCA) to assess face validity and again after being placed online utilizing Qualtrics. This study was approved by the Human Subjects Review Board of Colorado State University (20-10370H). Data were analyzed with IBM SPSS Statistics 26 statistical software.

The two programs assessed included Asheville Humane Society (AHS) mobile veterinary care clinic (targeting all clients who accessed services between 11/2017–11/2019), and Asheville Humane Society (AHS) Affordable Pet Care Clinic (targeting all clients who accessed services between 3/1/18–3/1/20). Asheville Humane Society mobile veterinary care clinic (MVC) consisted of one veterinarian, one veterinary technician, and two AHS staff members and provided services to underserved communities approximately twice a month. The unit typically spent 2–3 h in one community before traveling to the next; visiting three communities during each outing and servicing ~9 owners and 12 pets at each location. The type of services the unit provided included injury care, illness visits, vaccinations, wellness exams, and medical grooming. Pet owners were seen on a walk-in basis with a typical wait time of ~15–30 min. A Spanish-speaking AHS staff member was present to translate, and all services were provided free of charge.

Asheville Humane Society Affordable Pet Care Clinic (APCC) was originally housed in the AHS adoption center, and then moved to a nearby veterinary clinic until it was permanently relocated to the new AHS community center building in 2019. The clinic was open once a month and offered reduced-cost services including illness and injury-related care as well as vaccinations and wellness exams. Clients were seen on a walk-in basis, with typical wait times of ~30 min. The clinic typically served 11-12 clients and 20 pets with a staff that consisted of one local veterinary hospital's veterinarian, veterinary technician and additional staff member as well as 3–4 AHS staff members. A Spanish-speaking AHS staff member was on hand to translate. Patient transportation to and from the clinic was provided upon request. Due to liability, APCC was not able to provide owner transportation. Owners could join their pet by finding other means of transportation or remain at home and receive updates and communication as needed during and after the appointment via telephone or text message.

## Results

A total of 64 surveys were completed by pet owners who accessed the APCC program (42.7% of the total number (*n* = 150) of APCC users between 3/1/18–3/1/20). The respondents included 45 (71.4%) dog owners, 17 (27.0%) cat owners and 1 (1.6%) owner who reported owning both a cat and dog. For the MVC survey, a total of 33 responses were received (24.4% of the total number of MVC users, *n* = 135, between 11/2017–11/2019). These users included 23 (69.7%) dog owners, 7 (21.2%) cat owners and 3 (9.1%) owners who reported owning both a cat and dog. The majority of pet owners utilizing these programs were White, non-Hispanic females with annual household income of < $20,000 (Tables 1, 2). For owners utilizing the MVC, the percentage of unemployed owners increased from 29% pre-COVID-19 to 42%. Similar results were seen for APCC clients; the unemployment rate for these clients pre-COVID-19 was 29% compared to 37% at the time of the survey. A substantial number of respondents accessing these programs reported being unemployed, with lower numbers of unemployment reported prior to the COVID-19 pandemic (Table 2). The mean age of respondents from the MVC survey was 48.3 (SD = 14.0; range = 20–73), similar to the mean age of owners from the APCC survey of 49.6 (SD = 14.0; range = 19–78).

**Table 1:** Demographics of pet owners utilizing services at APCC and MVC.

	**APCC *n* = 59**	**MVC *n* = 31**
**Gender**		
Male	18 (30.5%)	5 (16.1%)
Female	38 (64.4%)	22 (71.0%)
Other	1 (1.7%)	2 (6.5%)
Prefer to not answer	2 (3.4%)	2 (6.5%)
	**APCC** ***n =*** **59**	**MVC** ***n =*** **30**
**Ethnicity**		
Hispanic or Latino	4 (6.8%)	2 (6.7%)
Not Hispanic or Latino	53 (89.8%)	25 (83.3%)
Prefer to not answer	2 (3.4%)	3 (10.0%)
	**APCC** ***n =*** **59**	**MVC** ***n =*** **31**
**Language most comfortable speaking**		
English	57 (96.6%)	30 (96.8%)
Spanish	1 (1.7%)	–
Prefer to not answer	1 (1.7%)	1 (3.2%)
	**APCC** ***n =*** **59**	**MVC** ***n =*** **31**
**Race**		
American Indian or Alaska Native	1 (1.7%)	–
Black or African American	5 (8.5%)	9 (29.0%)
Hispanic or Latino	2 (3.4%)	1 (3.2%)
White	49 (83.1%)	19 (61.3%)
Other	2 (3.4%)	-
Prefer to not answer		2 (6.5%)

**Table 2:** Employment status and income of pet owners utilizing services at APCC and MVC.

	**APCC** ***n =*** **59**		**MVC** ***n =*** **31**	
	**Current**	**Right before** **COVID-19**	**Current**	**Right before** **COVID-19**
**Employment status**				
Full time	10 (16.9%)	11 (18.6%)	4 (12.9%)	8 (25.8%)
Part time	8 (13.6%)	14 (23.7%)	4 (12.9%)	5 (16.1%)
Unemployed	22 (37.3%)	17 (28.8%)	13 (41.9%)	9 (29.0%)
Retired	16 (27.1%)	15 (25.4%)	7 (22.6%)	6 (19.4%)
Prefer to not answer	3 (5.1%)	2 (3.4%)	3 (9.7%)	3 (9.7%)
	**APCC** ***n =*** **59**		**MVC** ***n =*** **31**	
**Total annual household income**				
Less than $20,000	39 (66.1%)		24 (77.4%)	
$20,000–$44,999	9 (15.3%)		3 (9.7%)	
$45,000–$74,999	3 (5.1%)		1 (3.2%)	
Prefer to not answer	8 (13.6%)		3 (9.7%)	

### Reasons for Most Recent Visit

The most common reason for owners' last visit to both the MVC and the APCC was to obtain preventative care (APCC = 50%, MVC = 69.7%), followed by sick care that are not emergencies (APCC = 40.6%, MVC = 27.3%). When asked about previous veterinary care, over half of owners visiting the MVC indicated their pet had never received veterinary care (54.5%). This number was slightly lower for those accessing the APCC (42.9%) (Table 3).

**Table 3:** Stated reasons for most recent visit to APCC and MVC and report of previous veterinary care.

	**APCC *n =* 64**	**MVC *n =* 33**
**REASON FOR MOST RECENT VISIT**		
**Preventative**		
Vaccinations	30 (46.9%)	21 (63.6%)
Wellness exam	18 (28.1%)	9 (27.3%)
**Sick Care**		
Illness	24 (37.5%)	9 (27.3%)
Injury	2 (3.1%)	1 (3.0%)
Other (fleas)	6 (9.4%)	5 (15.2%)
Other	9 (14.1%) (eye or ear problems, stitch removal, hair loss, allergy, anal glands, microchip, deworming)	5 (15.2%) [microchip, nail trim (2), anal glands, dog needed home]
Don't know/remember		1 (3.0%)
	**APCC** ***n =*** **63**	**MVC** ***n =*** **33**
**PREVIOUS VETERINARY CARE**		
Yes—in the previous 3 years	31 (49.2%)	12 (36.4%)
Yes—but not in the previous 3 years	4 (6.3%)	2 (6.1%)
No	27 (42.9%)	18 (54.5%)
Don't know/don't remember	1 (1.6%)	1 (3.0%)

### Perceptions of Veterinary Care Experience

**Figure 1:**
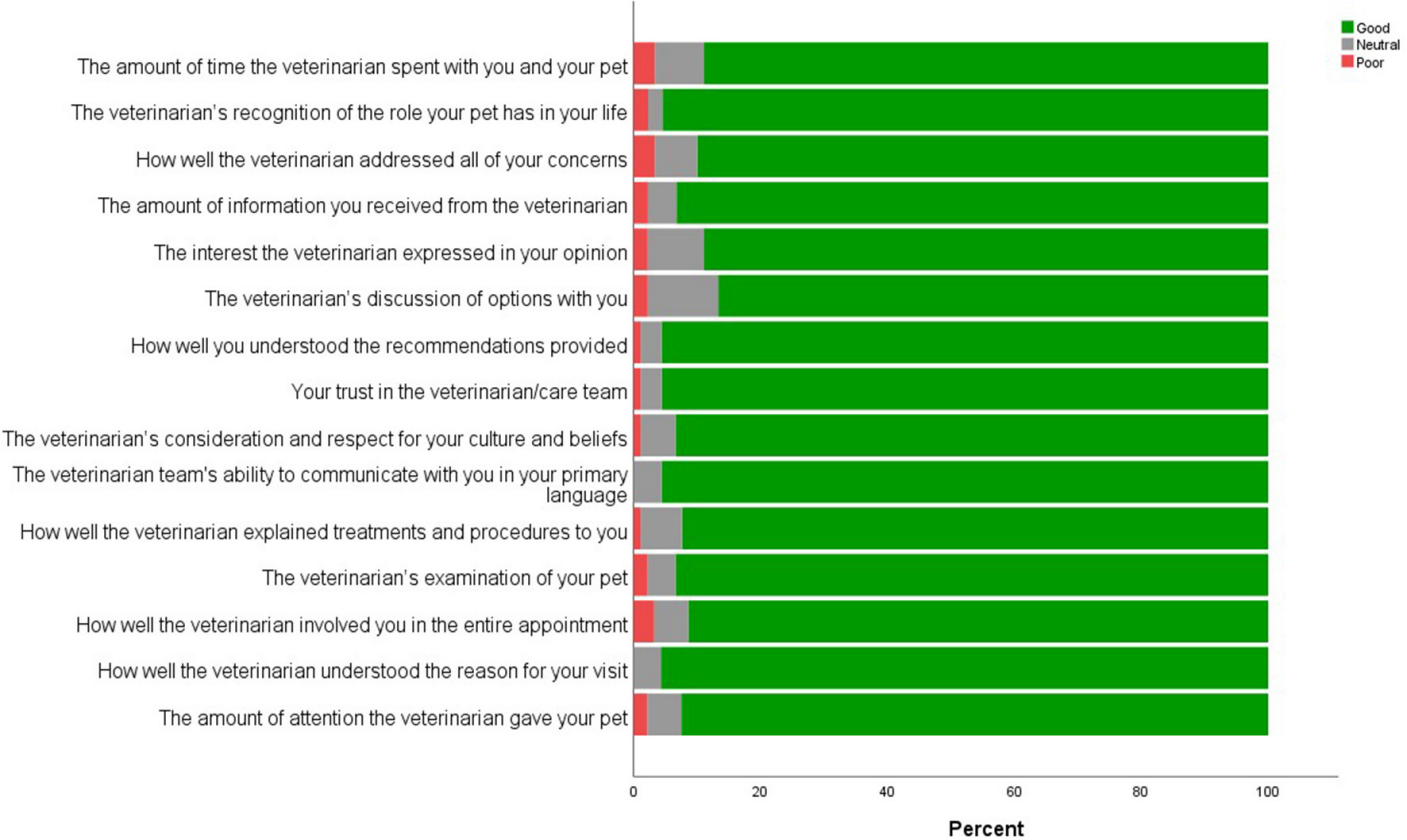
Rating of most recent veterinary visit by pet owners utilizing services at the APCC and MVC (excluding don't know responses).

Pet owners accessing care from both the MVC and the APCC programs were asked several questions about their most recent veterinary experience (Table 4 and Figure 1). The majority of owners rated both programs highly positive. The areas that received the lowest relative ratings for the MVC program included how well the veterinarian explained treatment and procedures, and the amount of time they were able to spend with the veterinarian—yet both of these areas were rated “good” (the most positive response) by over 80% of respondents. The areas that received the lowest scores for the APCC program, relative to other areas, included questions pertaining to the interest the veterinarian expressed in their opinions, the discussion by the veterinarian about treatment options and the cost/charges for the visit. All of these areas, however, were rated “good” by over 80% of respondents (Figure 1). Owners were also asked how important the veterinary care they received was in helping them keep their pet. For both programs, the majority of owners reported that the care they received was very important (APCC: very important = 86.9%, moderately important = 9.8%, not important = 3.3%; MVC: very important = 87.5%; not important = 12.5%).

**Table 4:** Rating of most recent veterinary visit by pet owners utilizing services at the MVC and APCC.

	**Agree**		**Neutral**		**Disagree**		**DK**	
	**MVC**	**APCC**	**MVC**	**APCC**	**MVC**	**APCC**	**MVC**	**APCC**
I felt respected by the staff	31 (97%)	59 (98%)	0	1 (2%)	0	0	1 (3%)	0
I believe the staff genuinely care about me and my pet	32 (97%)	58 (97%)	0	2 (3%)	1 (3%)	0	0	0

To assess barriers to accessing veterinary care, participants were asked to indicate what challenges they had obtaining the care they needed. For the APCC, the most common barrier was financial, followed by transportation issues while the most common barrier noted by MVC clients pertained to uncertainty about the mobile unit's schedule and hours (Table 5).

**Table 5:** Pet owners' perceived barriers to accessing veterinary care at APCC and MVC.

**Program**	**APCC *n =* 64**	**MVC *n =* 33**
Unsure of clinic schedule/hours	13 (20.3%)	9 (27.3%)
Inability to get time off from work/school	4 (6.3%)	3 (9.1%)
Hours/days the Affordable Pet Care Clinic is open	7 (10.9%)	2 (6.1%)
Language barrier	0	0
Did not have the money	19 (30.0%)	Not applicable
Transportation problems	14 (21.9%)	Not applicable
Don't know	0	1 (3.0%)
Other	6 (9.4%) (ability to make appointment, childcare, miscommunication, COVID-19-related issue)	5 (15.2%) (homebound, owner mobility issues, safety concerns, unsure about location.

## Discussion

Pets are seen as important members of the family for many, offering happiness, comfort and support. Yet, many families struggle to provide their pet the veterinary care they need to stay healthy. Even before the COVID-19 pandemic, it was estimated that 23 million pets live with impoverished families (26) and this problem has only been exacerbated with the pandemic (27). Further, numerous shelters have reported substantial adoption rate increases as people have been spending more time at home. The financial ramifications of COVID-19, as evidenced in the current study, have not impacted all groups of people equally; unemployment rates for young Black and Hispanic men and women, for example, are much higher than that for young White men and women (28). Employment changes due to COVID-19 can be seen within this study's sample.

While some might propose that people without the financial means to care for a pet should not have one, we argue that it is not acceptable, nor ethical, to deny families the option to have a pet due to barriers in accessing veterinary health care. As noted by Wiltzius et al. suggesting that people with limited means should not have pets is an “untenable solution” (21). Additionally, with research suggesting that pets play a critical role in mitigating COVID-19 related stress (29–31), and numerous antidotal reports that pet adoptions have dramatically increased since the pandemic began, it is more critical than ever to support these vulnerable pet owners. Assisting these families that struggle with financial constraints that prevent them from accessing veterinary care could help them keep their pets at home (20).

To address these barriers, many communities have created a variety of low-cost or free veterinary programs. Yet, not everyone is supportive of such programs. There is a common misconception that providing discounted or free veterinary services will take paying clients away from nearby veterinary hospitals, despite the fact that studies have consistently shown that the majority of owners accessing these services have not seen a veterinarian before (21, 32). Our study found similar results, whereby 55% of pets seen through the MVC and 43% seen at the APCC had never been to a veterinarian before.

While cost is the most common barrier to veterinary health care, as well as a determinate factor in relinquishment decisions (20, 33, 34), financial limitations are not the only barrier to veterinary care that must be addressed. Accessibility and transportation are additional key components that must be considered to ensure pets receive the care they need. It is not enough to make veterinary care affordable; it must also be easily accessible. Transportation is a potential barrier that poses unique challenges for veterinary care. Owners without a car may be forced to rely on public transportation that does not allow animals. Alternative options, such as private transportation in the form of taxis or Uber rides, if available in their community, can be cost prohibitive. This lack of transportation can be a challenge even if a veterinary clinic is just a few miles away. There is a need for veterinary services to come to these owners in the form of mobile clinics.

Regardless of the type of low-cost or free community veterinary program created, it is imperative that it offers a positive experience for the pet owner. This involves attention to communication and cultural competence. Good communication between veterinarians and pet owners is critical and has been shown to lead to better clinical outcomes, higher client satisfaction, and increased compliance with recommended care (35–38). While traditionally veterinarian-client relationships were more paternalistic, a “relationship-centered” approach has more recently been promoted. This approach is one of balanced power between the client and veterinarian and is based on mutuality, negotiation and joint agreement (39–41). Recent studies investigating veterinary communication have suggested that clients prefer a collaborative partnership with their veterinarians; one in which they are involved in the decision-making process (42, 43). Clients want their veterinarians to take time to listen to them and clearly explain diagnoses and recommendations (44). In addition to being heard, clients want their veterinarian to be empathic; defined as the ability to take the perspective of the client and demonstrate compassionate care (45). The ability to convey empathy has been shown to help develop rapport, establish trust, and increase compliance; all of which lead to better patient outcome (46–48).

A key component of a mutually respectful collaborative relationship includes cultural competence, which can be defined as awareness, behaviors, knowledge, attitudes, skills, and policies that all come together to enable people to work effectively in cross-cultural situations (45, 49). Demonstrating cultural competence when communicating with clients invites mutuality and inclusion, leading to higher client satisfaction and improved animal health (49). While enhanced cultural competence cannot compensate for other barriers to veterinary care, it can help. Pet owners who feel respected and heard are more likely to seek out care and follow medical recommendations (49).

Effective communication, empathy, and cultural competence are all needed to ensure a positive client experience. We suggest that it is not enough to offer low-cost community veterinary programs that do not excel in all of these critical areas. Given the fact that the majority of pet owners utilizing these community resources have limited prior veterinary medicine experience, it is imperative that these experiences are positive to enhance the chances that they will seek out subsequent veterinary care. This study is one of the first to assess these critical components. The results from this study suggest that it is possible to create low-cost community programs that satisfy clients' needs to be heard, valued and respected. For example, over 90% of clients from both programs reported feeling their veterinarian respected their culture/beliefs and recognized the role their pet played in their lives. Most owners also reported feeling their veterinarian wanted to hear their opinion, discussed options and recommendations, and included them in the entire veterinary visit. Additionally, over 85% reported trusting the veterinary team and feeling the community service was important in helping them keep their pet. With such positive experiences, it seems likely that the groundwork has been laid for these owners to access available and accessible veterinary care services in the future.

Limitations to this study include the fact that it focused on only community programs and the survey was not available in Spanish. Further study on additional programs, as well as views from all entities involved, is suggested. In summary, there is a growing need to assist the number of families who cannot access veterinary care. Low-cost or free community programs offer one potential solution, but care must be taken to ensure the experience is a positive one for all involved. Careful attention to communication, empathy and cultural sensitivity are critical in ensuring the success of such programs. In this way, these programs create a positive foundation for future veterinary care and a positive ongoing relationship between pet owners, their pets, and veterinary professionals.

## Data Availability Statement

The raw data supporting the conclusions of this article will be made available by the authors, without undue reservation.

## Ethics Statement

The studies involving human participants were reviewed and approved by Colorado State University Regulatory Compliance Committee. Written informed consent for participation was not required for this study in accordance with the national legislation and the institutional requirements.

## Author Contributions

LK analyzed the data. All authors conceived the study design and involved with the manuscript writing and editing.

## Conflict of Interest

The authors declare that the research was conducted in the absence of any commercial or financial relationships that could be construed as a potential conflict of interest.
